# Materials and Devices for Biodegradable and Soft Biomedical Electronics

**DOI:** 10.3390/ma11112108

**Published:** 2018-10-26

**Authors:** Rongfeng Li, Liu Wang, Lan Yin

**Affiliations:** School of Materials Science and Engineering, The Key Laboratory of Advanced Materials of Ministry of Education, State Key Laboratory of New Ceramics and Fine Processing, Tsinghua University, Beijing 100084, China; luckylrf@163.com (R.L.); liuwang@mail.tsinghua.edu.cn (L.W.)

**Keywords:** biodegradable electronics, transient electronics, soft biomedical electronics, biodegradable materials

## Abstract

Biodegradable and soft biomedical electronics that eliminate secondary surgery and ensure intimate contact with soft biological tissues of the human body are of growing interest, due to their emerging applications in high-quality healthcare monitoring and effective disease treatments. Recent systematic studies have significantly expanded the biodegradable electronic materials database, and various novel transient systems have been proposed. Biodegradable materials with soft properties and integration schemes of flexible or/and stretchable platforms will further advance electronic systems that match the properties of biological systems, providing an important step along the path towards clinical trials. This review focuses on recent progress and achievements in biodegradable and soft electronics for biomedical applications. The available biodegradable materials in their soft formats, the associated novel fabrication schemes, the device layouts, and the functionality of a variety of fully bioresorbable and soft devices, are reviewed. Finally, the key challenges and possible future directions of biodegradable and soft electronics are provided.

## 1. Introduction

With the growth of the global economy, and the development of science and technology, a massive assortment of electronics has been widely used in human society, which plays an important role in industrial processes, telecommunication, entertainment, healthcare, etc. [1,2,3,4,5]. Soft electronics that ensure conformal contact with nonplanar surfaces, such as soft biological tissues, are expected to play crucial roles in healthcare. The characteristic of these electronics is that they can significantly expand the capabilities of conventional rigid electronics in sensing, monitoring, diagnosing, and potentially intervening functions. The intimate contact between the soft device and the nonplanar object allows for high-quality data to be collected. Additionally, in the area of medical devices, soft electronics have similar mechanical properties to biological tissues and, thus, they cause minimal irritation to the human body.

On the other hand, biodegradable electronics possess unique characteristics and attract numerous research interests. The devices can dissolve, resorb, or physically disappear into physiological or environmental solutions, partially or completely, at controlled rates after the expecting working period [6,7,8,9,10,11,12,13,14]. Although long-lasting operation is one hallmark of traditional electronics, devices with biodegradability can potentially offer great benefits for temporary biomedical implants, green environmental electronics, and secured hardware. Biodegradable electronics can serve as temporary diagnostic and therapeutic platforms for important biological processes, e.g., wound healing and tissue regeneration, and they can be safely resorbed by the body after usage, eliminating a second surgery for device retrieval, and therefore avoiding associated infection risks and hospital costs [15]. Biodegradable electronics also provides an alternative way to alleviate issues that are associated with electronic waste (e-waste) [16], and they enable potential usage for security hardware, preventing unauthorized access of personal or security information [17,18].

Serving as medical implants, biodegradable electronics eliminate the potential retention of device materials, while soft electronics ensure conformal wrapping with the human body, as they are soft, curvilinear, and evolving [19]. Recent research on advanced materials [14], fabrication approaches [20], and design layouts [21] yield biodegradable and soft electronics that enable intimate integration into the body, with unique capabilities for diagnostic and therapeutic functions, which would otherwise be impossible when using conventional wafer-based electronics that are built upon non-degradable and rigid printed circuit boards. These emerging technologies provide critical tools that could have great potential to improve human health and enhance the understanding of biological systems.

This review specifically focuses on recent progress and achievements in electronics that combine both soft and biodegradable characteristics, targeting biomedical applications, while reviews on general transient electronics can be found elsewhere in references [6,13]. The available biodegradable materials in the soft formats, and their associated fabrication schemes are first reviewed, followed by the introduction of device layouts and the functionality of a variety of fully bioresorbable and soft devices, including solutions for power supply. Perspectives and the outlook of biodegradable soft electronics for clinical medicine are also provided at the end of the article.

## 2. Materials

A wide range of biodegradable materials have been explored to build biodegradable electronics. Traditional biodegradable materials are mostly based on polymers and magnesium alloys, and they serve mainly as structural components, e.g., cardiovascular stents and 3D scaffolds. As electronic properties are essential for constructing electronics, dissolvable inorganic materials with excellent operational characteristics are therefore also of great interest.

In addition to biodegradability, soft characteristics are another critical property to be considered for biomedical applications, in order to achieve minimal irritation to the body, and to obtain intimate contact with biological tissues. Soft materials should be able to survive mechanical deformations, and simultaneously, their functional properties should remain unaffected. The term “soft” can refer to flexible, foldable, stretchable, and twistable, and here, flexible materials are the focus.

The basic building blocks for electronic components are semiconductors, dielectrics, and conductors, and studies have developed strategies to ensure flexibility. The key method is to configure biodegradable inorganic semiconductors, dielectrics, and metals into thin film and open mesh formats, and to integrate them onto soft biodegradable polymeric or metal foil substrates. Through these techniques, biodegradable and flexible electronics that can adapt well to the soft nature of the human body.

In the following sections, inorganic biodegradable functional materials, substrate materials, and encapsulation materials, and organic functional materials will be reviewed respectively, in terms of their respective dissolution rates, mechanical properties, and biocompatibilities. Dissolution data from the literature of major inorganic materials are summarized in tables for better comparison. In all, dissolution rates of a wide range of biodegradable materials have been investigated in detail in simulated bio-fluids, such as phosphate-buffered saline (PBS), Hanks’ solutions, artificial cerebrospinal fluid (ACSF), etc. A few studies have investigated the effects of proteins on the dissolution rates of Si. Strategies have been proposed to obtain soft biodegradable materials that combine both inorganic and organic components. Although detailed studies are needed to further reveal the pertinent biological influences, biodegradable materials of interest exhibit good biocompatibility through their evaluation in cell studies and animal trials.

### 2.1. Inorganic Functional Materials

Functional materials are key components for electronics, and they consist of semiconductors, conductive materials, and dielectric materials. Inorganic dissolvable thin-film materials that have been explored, include monocrystalline silicon (mono-Si), polycrystalline silicon (poly-Si), amorphous silicon (a-Si), germanium (Ge), silicon germanium alloy (SiGe), indium–gallium–zinc oxide (a-IGZO), and zinc oxide (ZnO) [22,23,24,25,26,27] for semiconductors; magnesium (Mg), molybdenum (Mo), tungsten (W), iron (Fe), and zinc (Zn) for conductive materials [23,27,28,29]; and magnesium oxide (MgO), silicon dioxide (SiO_2_), and silicon nitride (SiN_x_) for dielectric materials [14,20,30]. The acceptable levels of these elements can be informed from nutritional supplements. The recommended dietary allowance and tolerable upper intake levels of functional materials are summarized in Table 1. As is shown, Mg, Mo, Fe, and Zn are all necessary elements for the human body. It should be noticed that the mean intakes of Si in adult men and women are 40 and 19 mg day^−1^ respectively, and limited toxicity research on Si suggests that there is no risk of inducing adverse effects for the general population, based on the common intake level [31].The mean total Ge exposure for people is 4 μg day^−1^, which can be absorbed from the intestinal tract and excreted largely through the kidneys [32,33,34]. In addition, W is usually found in rice, with concentrations of 7–283 μg kg^−1^ [35]. However, because of a lack of adequate and sufficient data for Si, Ge, and W, it is necessary to establish a recommended dietary allowance (RDA) and tolerable upper intake levels (UL).

Functional materials in the thin film format are adopted to assure flexibility, as well as reasonable degradation time frames. Studies have revealed that nanomembrane materials have high degrees of bendability, as the flexural rigidity and energy release rates scale down with thickness, enabling an intimate contact with non-planar and curvilinear surface [37]. Nanomembranes can be obtained by peeling the top membrane materials from commercially available wafers (e.g., silicon on insulator, SOI), which will be discussed later. Low-temperature deposition such as radiofrequency plasma-enhanced chemical vapor deposition (RF-PECVD) [38], electron cyclotron resonance (ECR) [39], and hot-wire chemical vapor deposition (HW-CVD) [40] can be used to fabricate high-quality functional thin films directly onto soft substrates. Additionally, solution-printing techniques have also been proposed as a low-cost alternative methods [41]. Another robust strategy for achieving soft materials is through structural design, which not only enhances the flexibility of intrinsic soft materials, but also gives flexibility to materials that are intrinsically rigid. Methods include separating rigid thin film materials into small islands, introducing serpentine, wavy, buckled interconnects, integrating rigid materials with soft substrates, etc. [42,43].

The research on silicon nanomembrane (Si NM) dissolution behavior greatly promotes the development of transient electronics, as it can leverage existing well-established Si semiconductor technology and realize high-performance biodegradable electronics. The dissolution rates of Si NMs in solutions with different ionic types and relevant concentrations [23,24], temperatures [24], pH values [23], concentrations of protein [44], as well as doping levels [22] have been investigated, all of which play an important role during the silicon dissolution process. The dissolution rates of Si NMs under different conditions are tabulated in Table 2. For example, dissolution rates have been observed for Si NMs (slightly p-doped 10^−17^ cm^−3^, 100 orientation) in aqueous solutions containing various chloride and phosphate concentrations at different temperatures. Higher temperatures and concentrations of chlorides and phosphates can greatly promote Si dissolution, probably through a nucleophilic dissolution process [24]. The underlying mechanism regarding the influence of chlorides and phosphates has been evaluated through density functional theory (DFT) and molecular dynamics (MD) simulations [24]. Dissolution rates of Si are found to be sensitive to calcium and magnesium ions as well [44]; e.g., the addition of 1 mM of Ca^2+^ and Mg^2+^ can slightly increase the rates in phosphate buffered saline solutions. As shown in Table 2, the presence of albumin decelerates the dissolution rates, probably due to the absorption of the protein onto the Si surface [44]. In addition, the types and concentrations of dopants for Si NMs can affect the dissolution rate significantly, and a sharp decrease in dissolution rate can be found when dopant concentrations exceed a certain level, i.e., 10^20^ cm^−3^.

Similarly, dissolution rates of poly-Si, a-Si, alloys of silicon, SiGe, and Ge show great dependence on the pH, temperatures, proteins, and type of ions [26]. For instance, the rates of these materials at physiological temperatures (37 °C) are higher than those at room temperature. At similar pHs, bovine serum leads to dissolution rates at 37 °C that are 30–40 times higher than those of a phosphate buffer solution for poly-Si, a-Si, and nano-Si. For SiGe, the dissolution rate exhibits an even more strongly accelerated rate (~185 times) in bovine serum. Besides Si and SiGe, dissolution rates of Ge and two-dimensional (2D) MoS_2_ materials have also been evaluated in physiological solutions, and the dissolution rates are summarized in Table 3. The dissolution of monolayer MoS_2_ crystals in PBS occurs as a defect-induced etching progress, in which the grain boundaries dissolve first, followed by the crystalline regions. Moreover, the increased concentrations of Na^+^ and K^+^ accelerate the degradation, because the existence of Na^+^ or K^+^ leads to lattice distortions of MoS_2_ and the formation of Na_2_S.

Metals related to trace elements that are normally found in human body, such as Mg, Zn, W, Fe, Mo, and their oxides, are great candidates for interconnects and dielectric materials [23,29,46]. Strain-tolerant metallic thin films can be fabricated by electron-beam deposition, pulsed-laser deposition, or magnetron sputtering, following photolithography. Among them, Mg and Zn are utilized more frequently, owing to the easy processing property and better concentration tolerance for patients, which means lower costs and safer resorbable properties. However, the degradation rates of Mg and Zn are relative fast; metals with slower rates (W, Mo) are, therefore, more desirable if a longer life time is needed [29]. Fe thin films become rusted easily, and they are converted to iron oxides and hydroxides, which have extremely lower solubilities in neutral solutions, and slightly acidic environments are probably more desirable for inducing complete degradation [29].

Moreover, dielectric materials, including magnesium oxide (MgO), silicon dioxide (SiO_2_), silicon nitride (Si_3_N_4_), and spin-on-glass (SOG), are also dissolvable in aqueous solutions. The dissolution rates of these materials depend not only on pHs, temperatures, and ion concentrations, but also on the physical and chemical properties of the films, which are affected by the deposition condition [10,14,47]. For example, the dissolution rates of oxides deposited by electron-beam (e-beam) evaporation are 100 times slower compared to those deposited by plasma-enhanced chemical vapor deposition (PECVD). For nitrides, the dissolution rate of a low-pressure chemical vapor deposition (LPCVD) nitride is slower than that of a PECVD nitride. The degradation rates of metals and dielectric materials are summarized in Table 4 and Table 5, respectively.

**Figure 1:**
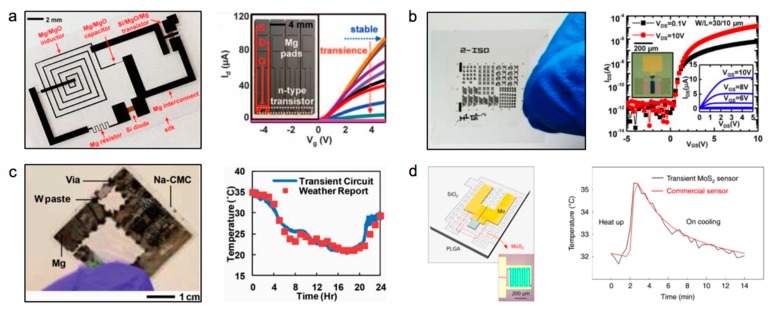
Different functional materials for soft and biodegradable devices. (**a**) Left: The circuit includes Si/MgO/Mg transistors, Si diodes, Mg/MgO inductors, and capacitors, as well as Mg resistors and interconnectors. Right: The transience of the operational characteristics of n-channel transistors. (**b**) Left: Schematic illustrations of transient Ga_2_O_3_/In_2_O_3_/ZnO thin film transistors (TFTs) and circuits. Right: Transfer characteristics for Ga_2_O_3_/In_2_O_3_/ZnO TFTs with widths (W)/lengths (L) (=30/10 μm). (**c**) Left: A transient printed circuit board (PCB) device with W and Mg for the temperature sensor; Right: Comparison for environmental temperatures measured by a transient circuit and the meteorological system. (**d**) Left: A multifunctional sensor using Mo and MoS_2_; Right: Measurement of the intracranial temperature with transient MoS_2_ and commercial sensors. Reproduced with permission from [14,27,28,45].

Figure 1a shows the representative flexible circuit based on dissolvable inorganic Si electronic materials on silk substrate, including transistors made by Si/MgO/Mg, diodes made by Si, and inductors and capacitors made by Mg/MgO, as well as resistor and connection wires made by Mg. The related transience properties in the operational characteristics of n-channel transistors are shown on the left side, which are comparable with transistors that are built with non-dissolvable materials. Logic circuits can be built based on the transistor unit cells, which provide a promising path to achieving soft and biodegradable multi-functional Si electronics [14]. Figure 1b illustrates a transient circuit composed of Ga_2_O_3_/In_2_O_3_/ZnO thin film transistors (TFTs), with transfer performance with widths (W)/lengths (L) (=30/10 μm). The output feature corresponds to a 0 to 10 V gate bias, with a step of 2 V, for 0 to 5 V drain bias (V_DS_) [27], which represents an alternative inorganic semiconductor for soft transient electronics. In Figure 1c, W powders are integrated onto a flexible sodium carboxymethyl cellulose (Na–CMC) substrate to print a temperature sensor circuit with a sensing performance that is closed to the weather report, which indicates an alternative method for quickly achieving biodegradable circuits [28]. Two-dimensional materials, such as MoS_2_, with attractive optical, electrical, and mechanical properties, have also been explored, to form biodegradable electronics. Figure 1d shows a transient pressure sensor that is integrated with Mo and MoS_2_ as the functional materials, which can be utilized to prepare a temperature sensor in vivo. Meanwhile, the literature suggests that MoS_2_ can gradually dissolve in PBS solution (pH = 7.4) at 75 °C, which may be adjusted by changing the grain size [45]. This investigation offers new insights incorporating ultrathin 2D materials for bioresorbable devices.

The biocompatibility of the materials and the products of their dissolution are important for applications in bioresorbable electronics. In vitro cytotoxicity studies on mono-Si, poly-Si, α-Si, SiGe, and Ge, with both neighboring stromal fibroblast cells and infiltrating immune cells, suggest that both of these materials and their dissolution products are biocompatible [26]. In addition, in vivo evaluations of Si NMs implanted into the subdermal regions of an albino, laboratory-bred strain of the house mouse (BALB/c mice), show that following five weeks of implantation, immunoprofiling of lymphocytes from the peripheral blood and draining lymph nodes revealed no significant differences in the percentages of CD4+ and CD8+ T cells for implanted animals and sham-operated controls, which suggests long-term immunological and tissue biocompatibility [23]. Furthermore, an in vitro assessment of cytotoxicity on a patterned array of Si NMs using cells from a metastatic breast cancer cell line (MAD-MB-231), and in vivo toxicity studies by implanting Si NMs on silk in the subdermal region of BALB/c mice, suggest this material is biocompatible, and that it has the potential to be used for long-term implantation [43]. Recently, in vitro cytotoxicity explored on 2D MoS_2_ films with L-929 cells and mouse fibroblast cells showed that there is no adverse effect on cell adherence and proliferation in vitro for 24 days. In vivo long-term cytotoxicity and biocompatibility studies with MoS_2_ layers implanting subcutaneously into BALB/c mice suggests that MoS_2_ does not cause any serious immunological or inflammatory reactions, and that it is, therefore, suitable for long-term biomedical use.

### 2.2. Organic Functional Materials

Conducting, semiconducting, and dielectric polymers are natural bridges between electronics and soft matter, because the vast chemical design space for polymers allows for the tunability of electronic, mechanical, and transient properties. A general strategy to create dielectric polymers is to incorporate high dielectric constant fills (e.g., SiO_2_, aluminum oxide (Al_2_O_3_), hafnium oxide (HfO_2_)) into a polymer matrix [12]. To circumvent the use of inorganic fillers, plant-based fibers (e.g., cotton, jute, bamboo, and banana fibers), sugars (e.g., glucose and lactose), and DNA and its precursors are promising natural polymers that intrinsically possess practical dielectric properties [52,53,54,55,56,57,58].

For conducting polymers, conjugated polymers that have been doped into a conducting state are used for device interconnectors and contacts. Common conducting polymers are polypyrrole (PPy), polyanniline (PANI), and poly(3,4-ethylenedioxythiophene) (PEDOT) [59]. One strategy for fabricating biodegradable conducting polymers is to blend conjugated polymers with biodegradable, insulating polymers. It has to be noted that these composites fabricated in this way are partially degradable, which means they are disintegrable, but the conductive polymers parts cannot be fully broken down to their monomers. Fully biodegradable conductive polymers might be obtained by conjugation breaking, but the conductivity of the materials is relatively lower than the partially degradable conductive polymers [12]. In addition, typical semiconducting polymers are polythiophenes (e.g., poly(3-hexylthiophene), P3HT), and diketopyrrolopyrroles (DPP) [60,61].

As with conducting polymers, blending has been utilized to generate partially degradable semiconducting polymers, e.g., by blending poly(3-thiophene methyl acetate) (P3TMA), a derivative of P3HT, with thermoplastic polyurethane (TPU) [62]. Fully degradable polymeric semiconductors have been achieved recently by introducing reversible imine bonds between DPP and p-phenylenediamine [63]. Conjugated molecules found in nature could also be utilized to build biodegradable electronics, including the natural dye indigo from the plants *Indigofera tinctorial* and *Isatis tinctorial* [64], natural pigment melanins [65,66], and β-carotene and anthraquinone derivatives [54,67].

### 2.3. Substrate Materials

Compared with functional materials, often with thicknesses of a few hundreds of nanometers, substrate materials with thicknesses at the micrometer scale contribute to the majority of the weight. As for soft electronics, substrate materials are a critically important consideration, because their mechanical properties can dominate that of the integrated system. Polymeric materials are often used as flexible substrate materials. Candidate materials need to be compatible with device fabrication processes, which usually involves high temperatures, water, and harsh solvents and, therefore, considerations of material properties centering around thermal stability and solvent compatibility are necessary. Degradation times, swelling rates, mechanical robustness, and the biocompatibility of the substrate materials are also critical to guiding the selection of substrates and, thus, achieving devices with controlled operational timeframes, as well as characteristics that match tissue environments. Although the properties of polymeric substrates vary greatly from material to material, these substrates tend to be flexible and biodegradable.

**Figure 2:**
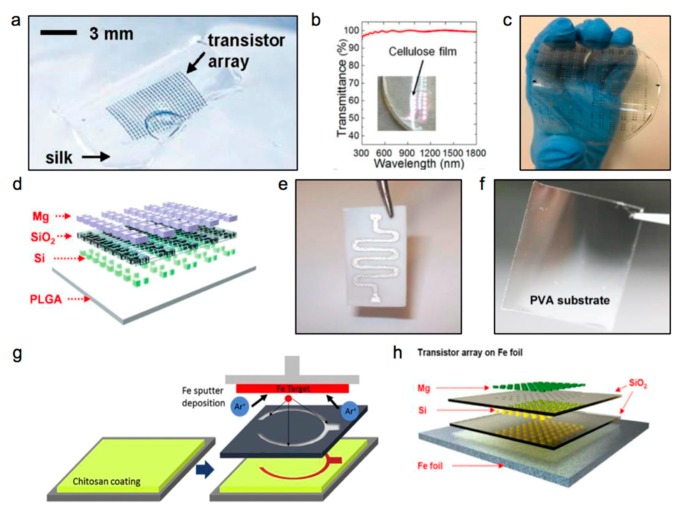
Various soft substrate materials for biodegradable electronics. (**a**) A silk substrate for transistor arrays. (**b**) A cellulose substrate for transistors. (**c**) A sodium carboxymethylcellulose (Na–CMC) bioresorbable substrate for Zn patterns. (**d**) A Poly lactic-co-glycolic acid (PLGA) substrate for transient electronic circuits. (**e**) An electrospun poly(caprolactone)–poly(glycerol sebacate) (PGS–PCL) sheet for a typical conductive pattern. (**f**) A PVA substrate for transient indium–gallium–zinc oxide (a-IGZO) TFTs. (**g**) A chitosan substrate for a biodegradable battery. (**h**) An Fe foil substrate for transistor arrays. Reproduced with permission from [20,27,47,63,85,86,87].

Based on these requirements, a series of substrate polymeric materials have been explored, which can be classified into natural materials and synthesized polymers. Materials that already exist in the natural environment have been applied as the substrates for biomedical electronics. These materials are biologically derived, which possess affinities and hypo-allergenicities to the human body, such as silk fibroin, cellulose, and chitosan, etc. Silk fibroin protein shows attractive properties that are related to biotechnology and biomedical fields [68,69,70,71,72], and possesses proper mechanical strength, a tunable life time, and minimum immune rejection. The n-channel metal-oxide-semiconductor field-effect transistors (MOSFETs) have been deposited onto thin silk film successfully, as shown in Figure 2a [20]. With similar characteristics, cellulose and chitosan are two other natural substrate materials, as shown in Figure 2b [63] and Figure 2g [73], respectively. As a type of polysaccharide, cellulose is a richly-abundant substance in nature that composes of more than one-third of all plant components, and it even reaches an abundance of 90% in cotton [74]. Cellulose is a promising substrate for biomedical implants, as it takes advantage of attractive biocompatibility properties and degradation in a physiological environment [75,76,77]. Recently, a novel transistor system using Al_2_O_3_ as the dielectric layer and Fe as the electrodes, was prepared on ultrathin cellulose film to fabricate transient electronics [63]. In addition, as a derivative product of chitin by the deacetylation process, chitosan is another common substrate for temporary devices with proven biocompatibilities, as shown in Figure 2g [73]. Moreover, some other natural compounds such as potato starch, gelatin, or caramelized glucose have been used as substrates for designing biodegradable electronics [54]. Generally speaking, the dominant advantages of natural substrate materials are favorable, in terms of low-immunoreaction and great abundance, as well as low cost.

However, the intrinsic properties of natural materials limit their applications, as biodegradable electronics draw higher demands for substrates with specific properties relating to stability, mechanical strength, and degradation rate, etc., which promotes research for synthetic polymers [12]. Synthetic polymeric materials have been used in numerous biomedical applications for years, such as in bioresorbable stents and sutures [78,79,80,81,82,83,84]. Poly lactic-co-glycolic acid (PLGA) is a typical polymer that is used as a transient substrate, which is a copolymer of poly lactic acid (PLA) and poly glycolic acid (PGA). Compared to natural materials, the lifetime and mechanical strength of PLGA can be modified over a wide range by adjusting the ratio of PLA and PGA, which paves a promising path for biomedical implants with controllable working lives. In Figure 2d, transient complementary metal-oxide-semiconductors (CMOSs) are prepared on PLGA substrates with excellent operational characteristics [85]. Because of the different substrate requirements for biodegradable clinical devices, numerous polymers with different mechanical and disintegration performances are prepared and utilized as elastic substrate layers for soft and transient electronics, such as sodium carboxymethylcellulose (Na–CMC) [86], poly(caprolactone)–poly(glycerol sebacate)(PGS–PCL) [87], and poly(vinyl alcohol) (PVA) [27], etc., as shown in Figure 2c,e,f, respectively. Among them, PVA is a water-soluble polymer that forms flexible layers, which allows for the controlled transport rate of water through altering the crosslinking density of the chains and their subsequent swelling ratios. A recent report suggests that biodegradable elastomer poly (octamethylene maleate (anhydride) citrate) (POMaC) is an excellent candidate for applications in terms of its biocompatibility, mechanical properties, and degradation characteristics, which can be tuned by varying the polymerization conditions [88]. Besides, poly(1,8-octanediol-*co*-citrate) (POC) is a biodegradable elastomer that can take strains up to ~30% with linear elastic mechanical responses. Hydrogels as hydrophilic polymeric networks with 3D microstructures represent alternative options that are highly biocompatible and suitable for biomimetic applications, due to their water-rich natures and structural similarities to natural extracellular matrices. Moreover, they can be designed to degrade under controlled modes and rates by enzymatic hydrolysis, ester hydrolysis, photolytic cleavage, or a combination of these reactions.

Although biodegradable polymeric substrates can often offer appropriate mechanical properties for soft and biodegradable electronics, direct device fabrication processed on polymer substrates is quite limited, as most types are sensitive to temperatures, solvents, or water. The swelling of most biodegradable polymers remains another challenge, as it can greatly shorten the functional lifetimes of electronic devices. Novel fabrication processes have been proposed to decouple polymeric substrates from the fabrication processes, which, however, introduce extra multiple fabrication steps; this will be discussed in the next section. Metal foils such as Mo, Fe, W, and Zn have also been proposed as alternatives to polymeric biodegradable substrates, to offer better compatibility with the fabrication process, because they are relatively temperature-resistant, and because they address swelling issues upon deployment in aqueous solutions, as illustrated in Figure 2h [47]. They can also show excellent electrical and thermal properties, favorable water and oxygen isolation performances, and they are relatively resistant to most solvents. However, the rigid properties of metal foils might limit their further applications.

### 2.4. Encapsulation Materials

Encapsulation materials, together with substrate materials, define the lifetimes of biodegradable electronics. Similarly, most polymeric substrate materials can be used to form strain-tolerant encapsulation layers, preventing rapid degradation of devices, such as PLGA, PCL, etc. [21,89]. Silk fibroin pockets have also been demonstrated to be useful for the controlled modulation of the device lifetime [90]. Similarly, POC can be used not only as the substrate layer, but also as the encapsulation layer, for transient biomedical devices, to protect them from the environment. However, water permeation resistance within biodegradable polymeric materials often cannot satisfy the requirements of devices when a longer lifetime is needed, e.g., an encapsulated Mg trace with silk fibroin could lose its conductivity within a few hours [14]. Encapsulation using a Si membrane (~1.5 μm) has been explored, and it can significantly extend the degradation times of dissolvable metals, e.g., Mg thin films with Si encapsulation result in a lifetime of 60 days in phosphate-buffered saline at 37 °C [44]. Bioresorbable electrocorticography electrodes based on Si encapsulation have demonstrated comparable recording results, compared to conventional standard electrodes, indicating the possibility of using a Si encapsulation layer for biodegradable electronics. In addition, alternating dielectric oxide layers of SiO_2_/Si_3_N_4_/SiO_2_ has also been proven to possess good water permeation resistance [30]. Further studies are needed to investigate biodegradable encapsulation materials with ultralow water permeation rates, as well as appropriate electrical properties and mechanical flexibilities, to achieve a wider range of operational time frames. Strategies include combining inorganic/organic multilayer materials to improve flexibility, and to introduce smart stimuli-responsive materials to precisely control the starting point of degradation.

## 3. Fabrication Schemes

Traditional device fabrication often involves photolithography, deposition, and etching processes. For the biomedical application, it is important to note that the implanted bioresorbable electronics require soft properties to realize their conformal contact with organs and tissues. In addition to that, the manufacturing process should not introduce any toxic materials or solvents, to guarantee favorable biocompatibilities. Consequently, novel fabrication techniques are needed to ensure material compatibility with the processing parameters.

For biodegradable devices, substrate materials with thicknesses at the micrometer scale contribute to the majority of the weight. Polymeric materials are often used as the substrates, because of their intrinsically soft and flexible properties, although metal foils have also been explored as an alternative [47]. Polymers are often dissolved into organic solvents and processed into a thin film format by drop casting, spin coating, or electrospinning methods. The thickness of the layer can be controlled by changing the relative solution concentrations, the speed of spin coating, or the electrospinning time [87,91]. The selections of proper solvents, spin-coating, or electrospinning parameters, and the surface treatments of handle substrates are critical to achieving free-standing polymer films with softness for the device fabrication.

**Figure 3:**
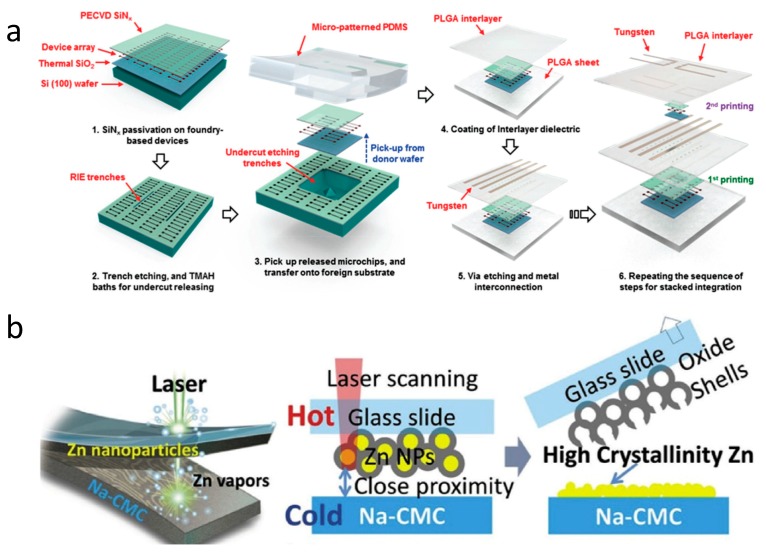
Fabrication schemes for soft and biodegradable electronics. (**a**) Preparation process of a 3D interconnected platform. Planarizing layers of PLGA serve as adhesives and interlayer dielectrics to facilitate 3D heterogeneous integration. (**b**) The schematic printing process for evaporation–condensation-mediated laser printing. Reproduced with permission from [21,86].

The direct deposition of functional materials onto biodegradable and soft substrates, has been achieved through the use of shadow masks [14]. Decoupling fabrication processes from target biodegradable substrates through transfer-printing enables devices with a higher level of integration and more versatile material selections [85]. Figure 3a shows a transfer printing process utilizing foundry-based devices to achieve biodegradable 3D heterogeneous integrated circuits [21]. In order to obtain releasable micro-components from foundry-base wafers, a ~700 nm SiN_x_ passive layer is deposited by plasma-enhanced chemical-vapor deposition (PECVD), followed by inductively-coupled plasma-reactive ion etching (ICP-RIE) process to form trenches. Poly(dimethylsiloxane) (PDMS) stamps are then utilized in a transfer printing process to remove and deliver the target collections (a total thickness of ~3 µm) onto target PLGA substrates. After that, a PLGA dielectric layer is prepared by the spin casting method, and a standard photolithography technology associated with RIE treatment, creates specific openings to make contact between the upper and lower layers. Another functional layer can then be prepared by applying a repeated procedure to finish the 3D integrated circuit. A combination of foundry-based Si wafers and transfer printing techniques provides a promising route towards high-performance and miniaturized biodegradable electronic systems.

Printing techniques of biodegradable materials represent alternative methods to realize quick circuit patterns [46,86,89,92]. Figure 3b illustrates a fabrication approach to manufacture bioresorbable electronics. A continuous-wave (CW) fiber laser is utilized to supply sintering power, so as to crystalize Zn nanoparticles onto a soft Na–CMC substrate directly, with superior conductivity (~1.124 × 10^6^ S m^−1^) [86]. Such a fabrication technology offers a direct way for a roll-to-roll preparation platform to produce soft biodegradable electronics with high integration levels and low costs. In addition, highly conductive bioresorbable inks with an extended lifetime, consisting of polyanhydride and dispersed molybdenum microparticles, has also been reported. Such an ink can be applied to flexible wire and connection joints, as well as the antennas of bioresorbable devices [92]. These novel printing processes imply a fast and low-cost way to achieve biodegradable circuits.

## 4. Representative Soft and Biodegradable Devices for Biomedical Applications

Compared to conventional rigid implants, soft properties are favorable for biomedical implants to ensure conformally wrapping around biosystems that achieve intimate contact and minimize mechanical irritations, which are crucial and necessary for their applications, such as physiological signal detection and drug delivery. Combining biodegradable characteristics, devices can achieve fully bioresorption after usage, eliminating device retrieval. These devices could potentially serve as implantable diagnostic and therapeutic platforms, and they provide unprecedented physiological information and treatments, which are especially valuable for temporal biological processes, such as wound healing, neural network mapping, drug delivery, tissue regeneration, etc. Demonstrated soft and biodegradable electronic implants and pertinent power supply solutions will be reviewed in the following sections.

### 4.1. Diagnostic Platforms

**Figure 4:**
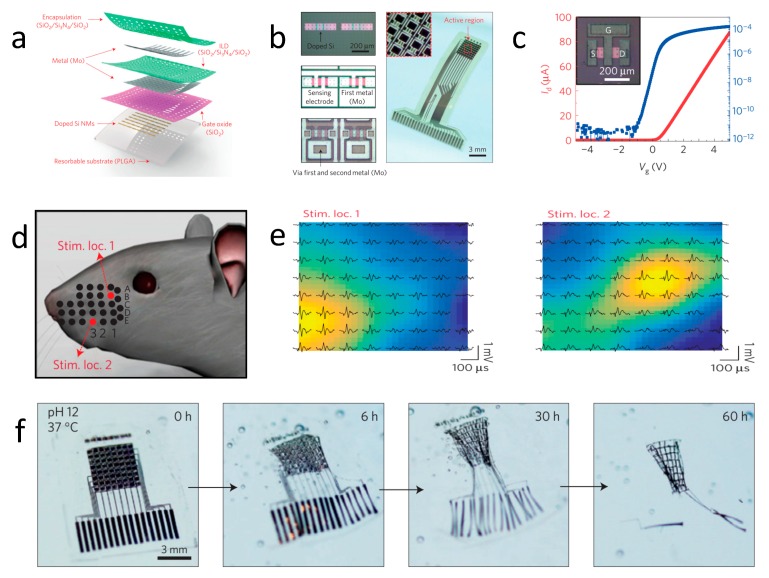
A soft and biodegradable neural electrode array sensor. (**a**) The schematic structure of an actively multiplexed sensing system for high-resolution electrocorticography. (**b**) Left: Optical micrograph images of a pair of subunits for the fabrication process. Right: The entire complete system. (**c**) Linear (red) and log scale (blue) transfer curves for a representative n-channel, MOSFET. (**d**) Schematic illustration of the whisker stimulation locations 1 and 2 (B1 and E3) in a rat model. (**e**) Left: Spatial distribution of the potentials evoked by the stimulation location B1. Right: Spatial distribution of the potentials evoked by stimulation location E3. (**f**) The degradation process at various stages of the sensor. Reproduced with permission from [30].

For diagnostic purposes, because of their close connection with tissues and organs, soft transient electronics can detect abnormal physiological signals sensitively and precisely, even at the early stages of specific diseases, before they can be observed by conventional equipment, which is important for human healthcare and follow-up therapy. Figure 4 shows a soft and high-resolution recording system for electrocorticography (ECoG) based on Si devices [30], which offers a potential utility for treating neural disorders where biodegradation is required, to avoid tissue injury upon device removal. The flexible platform also enables the intimate contact of the device with the cerebral cortex, and allows for high-fidelity data recording. Figure 4a illustrates the structure of the device, consisting of a flexible PLGA substrate (~30 μm), a Si nanomembrane semiconductor, a Mo electrode (~300 nm), a SiO_2_ gate dielectric, and SiO_2_ (~300 nm)/Si_3_N_4_ (~400 nm)/SiO_2_ (~300 nm) interlayer dielectrics. The device includes 128 metal oxide-semiconductor field-effect transistors (MOSFETs). Figure 4b exhibits the optical images of the unit cells of the device at different stages of fabrication, and a complete system. Figure 4c represents the linear (red) and log-scale (blue) transfer curves for representative n-channel MOSFET indication, with the mobility and on/off ratio of ~400 cm^2^ V^−1^ and ~10^8^, respectively. The device is implanted onto the whisker area, and Figure 4d reveals the schematic illustration of the whisker stimulation locations in a rat model. By stimulating the defined positions, the related evoked potential in a spatial distribution is recorded, as given in Figure 4e, indicating high resolution mapping of ECoG that matches or exceeds any existing devices. The sensing system can completely dissolve into an aqueous buffer solution gradually at pH = 12 and at 37 °C, as shown in Figure 4f. The sensitive sensing and biodegradable features of this transient device offer a promising application for internal physiological signal collection, which is significantly important for disease diagnosis.

**Figure 5:**
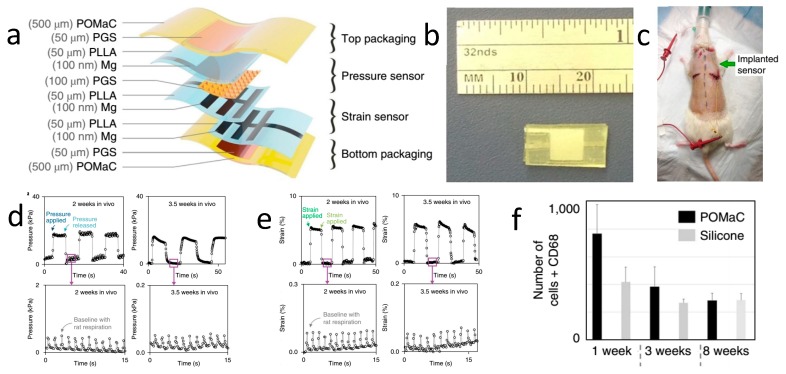
A stretchable and biodegradable strain and pressure sensor for orthopedic application. (**a**) Schematic diagram of the sensor space structure. (**b**) Optical image of the sensor. (**c**) The location of the implantable sensor. (**d**) Pressure signal detection two and 3.5 weeks after sensor implantation. (**e**) Strain signal detection two and 3.5 weeks after sensor implantation. (**f**) Results of immunohistochemistry. Reproduced with permission from [88].

Stretchable and biodegradable pressure and strain sensors have also been reported for real-time monitoring for potential tendon recovery [88]. Figure 5a,b show the vertical structure and optical images of the sensor, respectively. The entire sensor is divided into four parts, including bottom and top encapsulation layers, as well as strain- and pressure-sensing areas, with Mg as the electrode, POMaC as the stretchable packaging layer, Poly(lactic acid) (PLLA) as the substrate, and PGS as the stretchable dielectric layer. Such a design allows for independent measurements of the strain and pressure. The sensor is implanted onto the back of a Sprague Dawley rat, as shown in Figure 5c. Figure 5d,e illustrate the collected pressure and strain signals after implantation for 2 and 3.5 weeks; the similar curves indicate a stable working performance. The biocompatibility of the sensor is demonstrated in Figure 5f, with CD68 positive cells decreasing gradually as the implantation period extends, suggesting that the inflammatory reaction is mitigated and, therefore, that excellent biocompatibility is reached. After more than two weeks of implantation, this sensor begins to degrade gradually. This research demonstrates the potential use of biodegradable devices for orthopedic applications.

### 4.2. Therapeutic Devices

In addition to diagnostic functions, soft and biodegradable electronics could also play an important role in therapeutic processes. For therapeutic platforms, flexible and biodegradable devices can offer personalized and precision treatments based on controlled performance and degradation rates. Bioresorbable devices can combine with drug delivery vehicles to achieve controlled drug release systems, which are critical for disease treatments.

**Figure 6:**
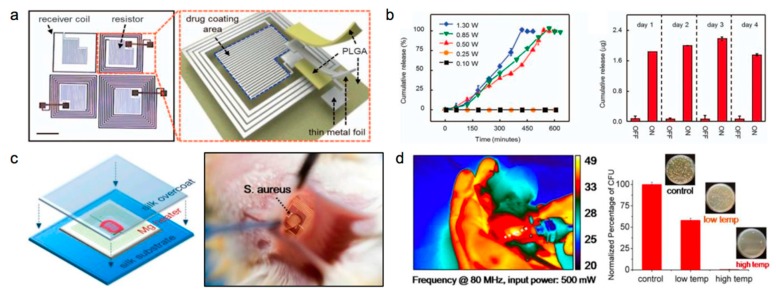
A thermally triggered drug delivery transient device and a radio frequency-controlled thermal therapy platform. (**a**) Images of the device structure. (**b**) Left: Cumulative release of doxorubicin from the device, operated with wireless power. Right: Controllable doxorubicin release from the device over 1 day on/off cycles. (**c**) Left: The schematic illustration of the radio frequency thermal therapy platform. Right: Photo of a device implanted in BALB/c mice. (**d**) Left: The thermal image of the device position while wirelessly powering the device. Right: The normalized number of colony-forming units after 24 h with different levels of input power. Reproduced with permission from [93,97].

The soft format of the sensor is favorable for controlled drug release with precise doses to specific areas, because of the conformal contact with tissues, which can improve treatment efficacy. Figure 6a displays the structure of a drug delivery system with a 2 × 2 array, consisting of inductive coupling coils and serpentine thermal heaters on a PLGA substrate [93]. The device allows for heating of the drug storage area through external wireless controls by inductive coupling. Drug release is thermally triggered by the phase transition of lipid layers imbedded with drugs. Figure 6b (left) shows the total cumulative percentage of doxorubicin released as a function of time from a device that is immersed in deionized water (12 mL) when it is activated by wireless external power between 0.1 to 1.3 W at 12.5 MHz and a distance of 2 mm. The release rate of the drug can be adjusted by adjusting the supplied power. Figure 6b (right) represents cumulative amounts of doxorubicin that are released wirelessly once a day, indicating good temperature-controlled drug release.

Wireless thermal therapy of bacterial management has also been demonstrated to assist with wound healing. Previous studies have revealed that some bacteria are highly sensitive to environmental changes, and that an increase in temperature lowers their survival [94,95,96]. Figure 6c (left) shows a transient radio frequency (RF) device for thermal therapy based on the Mg heater, embedded between silk fibroin layers [97]. Figure 6c (right) reveals related implantation processes for rats. The rats are infected by *Staphylococcus aureus* (*S. aureus*) with a subcutaneous injection (~5 μL) at the device implantation site, to mimic surgical site infections. The experimental rats are divided into untreated, low power (100 mW), and high power (500 mW) groups. Figure 6d (left) shows a thermal image of a rat with a high power supply after 10 min heat treatments, with the temperature reaching 49 °C. The infected tissues were collected after 24 h, and assessed by counting the normalized number of colony-forming units in the homogenates (n = 3) using standard plate-counting methods, and the related results are shown in Figure 6d (right). It is obvious that thermal treatment achieves a better bactericidal effect with higher temperatures. The concept of a biodegradable thermal therapy device can be widely applied to eliminate temperature-sensitive bacteria, which could be crucial to promoting the wound healing process.

### 4.3. Power Supply

Power supply is an essential component for biodegradable electronic systems, and great efforts have been made to explore biodegradable power sources, including batteries [98,99,100,101,102], supercapacitors [9], photovoltaic devices [103], radio frequency (RF) power scavengers [104], piezoelectric harvesters [25], etc. For biomedical applications, the power device should be flexible, stretchable, and miniaturized, to realize conformal contact and to minimize mechanical irritation.

**Figure 7:**
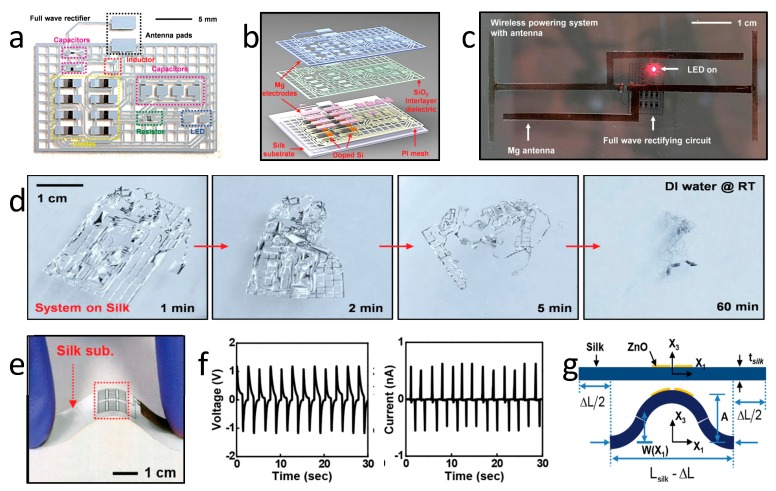
Flexible and biodegradable power harvesters. (**a**) A schematic illustration of transient RF power scavenging circuits. (**b**) A schematic illustration of an exploded view of the device. (**c**) An image of the device powered wirelessly with an RF transmitter and an Mg-receiving antenna. (**d**) Degradation process of the RF power harvester (**e**) Image of a soft and transient ZnO mechanical energy harvester on a silk substrate. (**f**) Output voltage and current ability during cycles of bending. (**g**) The theoretical shape for the buckling of a device under compression. Reproduced with permission from [25,104].

A flexible and biodegradable RF wireless energy harvester appears in Figure 7a–d. Figure 7a,b illustrates the schematic structure of a wireless RF power transmitter, which consists of an RF antenna, an inductor, six capacitors, a resistor, and eight diodes [104]. By integrating with an Mg antenna, the RF system is capable of transmitting enough power to light up a red LED, as shown in Figure 7c. The entire system degrades rapidly in deionized water, as shown in Figure 7d. The demonstrated system provides a route for wireless energy harvesting for biodegradable implants; however, the current device volume limits its usage, and it needs further improvement. Figure 7e exhibits a flexible and biodegradable piezoelectric energy harvester based on ZnO on a silk substrate [25]. By bending the integrated film repeatedly, an output potential of 1.14 V, and curves of 0.55 nA are obtained, as shown in Figure 7f. Figure 7g illustrates the theoretical shape for the buckling of a device under compression. Although the piezoelectric energy harvester does not rely on an external device such as that for an RF energy scavenger, mechanical deformation inside the body is limited and, therefore, this limits the available power that can be obtained.

In summary, although various power solutions have been proposed, the performance of soft and biodegradable power supplies still remains an obstacle. The power density and the working life of the device should be further improved to satisfy the requirements of various biodegradable electronic systems. Materials and fabrication methods for power devices should also be advanced to achieve miniaturized, flexible, and stretchable platforms that are suitable as biomedical implants.

## 5. Summary and Outlook

As an emerging field, soft and biodegradable electronics have attracted more and more research interest because of their foreseeable applications for clinical implants, eco-friendly devices, and security hardware. For biodegradable biomedical devices, favorable biocompatibility, appropriate degradable rates, and robust mechanical properties, as well as superior performance, are desirable. Many studies have been made, to achieve remarkable progress towards biomedical applications.

However, there are still many issues that need to be addressed. More versatile materials with both biodegradable and soft properties need to be explored, to further broaden potential applications. For example, biodegradable functional materials that are highly stretchable and flexible could expand suitable implantation locations and significantly improve data recording sensitivities and accuracies, and minimize the irritation and inflammation that are associated with implantation. This could be achieved by developing composite structures that integrate hard and soft components, as well as through appropriate mechanical structure designs. Moreover, encapsulation materials with superior water resistance and soft properties are critical for waterproof sealing, to avoid the potential rupture of the encapsulation layer causing water leakage. Different from the fabrication methods for rigid biodegradable devices, novel fabrication technologies should be further explored to produce soft electronics with low costs and easy manufacturing procedures, as well as high levels of integration. In addition, the performance of soft devices, such as fast response and excellent sensitivity, as well as high accuracy, needs further improvement, and multifunctional electronics should be fabricated to meet the requirements of clinical standards. A comprehensive investigation of the device/tissue interface, and the metabolic processes of degradation products are also necessary to clarify the safety issues of biodegradable devices. These studies will improve the overall performance of soft and biodegradable devices, and they will promote the development of transient electronics, which could potentially make disease diagnosis and treatments more precise, effective, and intelligent.

## Figures and Tables

**Table 1:** The recommended dietary allowance (RDA) and tolerable upper intake levels (UL) of biodegradable elements [31,36].

Life Stage	Category	Element			
		Mg (mg/day)	Mo (μg/day)	Fe (mg/day)	Zn (mg/day)
Infants 0–12 months	RDA	30–75	2–3	0.27–11	2–3
	UL	–	–	40	4–5
Children 1–8 years	RDA	80–130	17–22	7–10	3–5
	UL	65–110	300–600	40	7–12
Males ≥9 years	RDA	240–420	34–45	8–11	8–11
	UL	350	1100–2000	40–45	23–40
Females ≥9 years	RDA	240–320	34–45	8–15	8–9
	UL	350	1100–2000	40–45	23–40
Pregnancy 14–50 years	RDA	350–400	50	27	11–12
	UL	350	1700–2000	45	34–40
Lactation 14–50 years	RDA	310–360	50	9–10	12–13
	UL	350	1700–2000	45	34–40

**Table 2:** The dissolution behavior of silicon nanomembranes (Si NMs) under different conditions [22,23,24,26,44].

Functional Materials	Temperature °C	Aqueous Solution	pH	Doping Type cm^−3^	Dissolution Rate nm day^−1^
Mono-Si NMs [24]	37	Phosphate 0.05 M	7.5	10^17^(p)	2.512
		Phosphate 0.1 M			25.119
		Phosphate 0.5 M			50.119
		Phosphate 1 M			63.096
		Chloride 0.05 M			1.259
		Chloride 0.1 M			6.309
		Chloride 0.5 M			63.096
		Chloride 1 M			63.096
	50	Phosphate 0.05 M	7.5	10^17^(p)	3.162
		Phosphate 0.1 M			31.623
		Phosphate 0.5 M			125.893
		Phosphate 1 M			251.189
		Chloride 0.05 M			5.012
		Chloride 0.1 M			15.849
		Chloride 0.5 M			199.526
		Chloride 1 M			398.107
Mono-Si NMs [22]	37	Buffer solution 0.1 M	7.4	10^17^(p)	3.162
				10^19^(p)	3.162
				10^20^(p)	0.501
	37	Buffer solution 0.1 M	7.4	10^17^(b)	3.162
				10^19^(b)	3.162
				10^20^(b)	0.251
	Room temperature (RT)	Coke	2.6	-	0.600
	RT	Milk	6.4	-	23.300
	RT	PBS 0.1 M	7.4	-	1.820
Mono-Si NMs [23]	37	Bovine serum	7.4	-	100.800
		Sea water	7.8	-	4.115
Mono-Si NMs [44]	37	Albumin phosphate buffered saline (PBS) Na^+^	7.4	10^15^(b)	42.100
		Albumin PBS Mg^2+^			45.010
		Albumin PBS Ca^2+^			51.000
	20	Purified water	5.5	10^15^(b)	<0.010
				10^20^(b)	<0.010
	20	Tap water	7.5	10^15^(b)	0.710
				10^20^(b)	0.420
	37	Serum	7.4	10^15^(b)	21.020
				10^20^(b)	0.500
	37	Hank’s balanced salt solution (HBSS)	7.6	10^15^(b)	58.010
				10^20^(b)	7.010
	37	HBSS w/Ca, Mg	7.6	10^15^(b)	66.020
				10^20^(b)	8.020
	37	HBSS	8.2	10^15^(b)	129.030
				10^20^(b)	58.000
	37	HBSS w/Ca, Mg	8.2	10^15^(b)	178.000
				10^20^(b)	69.010
	20	Purified water	5.5	10^15^(b)	0.010
		Tap water	7.5		0.720
		Serum	7.4		3.500
	37	Purified water	5.5	10^15^(b)	0.210
		Tap water	7.5		2.620
		Sweat	4.5		0.530
		Serum	7.4		21.000
		HBSS	7.6		58.210
		HBSS w/Ca, Mg	7.6		66.010
		HBSS	8.2		129.020
		HBSS w/Ca, Mg	8.2		178.000
poly-Si NMs [26]	RT	Buffer solution	7	-	1.020
			7.4		1.585
			8		10.010
			10		398.107
	37	Buffer solution	7	-	1.259
			7.4		3.162
			8		19.953
			10		562.341
a-Si NMs [26]	RT	Buffer solution	7	-	1.778
			7.4		3.981
			8		15.849
			10		501.187
	37	Buffer solution	7	-	1.585
			7.4		5.011
			8		31.623
			10		630.957

Note: p stands for phosphate doping, and b stands for boron doping.

**Table 3:** The dissolution behavior of Ge and 2D MoS_2_ under different conditions [26,45].

Functional Materials	Temperature °C	Aqueous Solution	pH	Dissolution Rate nm day^−1^
Ge [26]	RT	Aqueous Buffer solution	7	0.794
			7.4	1.995
			8	15.849
			10	501.187
	37	Aqueous Buffer solution	7	1.259
			7.4	3.162
			8	19.953
			10	562.341
Poly-MoS_2_ [45]	40	1.0 M PBS	7.4	0.010
			12	0.010
	60	1.0 M PBS	7.4	0.070
			12	0.160
	75	1.0 M PBS	7.4	0.200
			12	0.270
	85	1.0 M PBS	7.4	0.270
			12	0.400

**Table 4:** Dissolution behavior of metals [47,48,49,50,51].

Functional Materials	Test Conditions	Dissolution Rate nm day^−1^	Dissolution Product
Mg [48]	Deionized water, RT	1680.000	Mg(OH)_2_
Zn [49]	Deionized water, RT	168.000	Zn(OH)_2_
Fe [47]	Simulated body fluids, 37 °C	5.000–80.000	Fe(OH)_2_, Fe(OH)_3_
Mo [50]	Deionized water, RT	7.200	H_2_MoO_4_
W [51]	Deionized water, RT	7.200–3.000–40.800	H_2_WO_4_

**Table 5:** Dissolution behaviors of dielectric materials in buffer solution at 37 °C [10,47].

Functional Materials	Fabrication Methods	Test Conditions	Dissolution Rate nm day^−1^	Dissolution Product
SiO_2_ [10]	E-beam	37 °C	10.000	Si(OH)_4_
	PECVD	37 °C	0.100	
	Thermally grown	37 °C	0.003	
Si_3_N_4_ [10]	LPCVD	pH 7.4	0.158	Si(OH)_4_ + NH_3_
	LPCVD	pH 8	0.251	
	LPCVD	pH 10	0.316	
	LPCVD	pH 12	0.631	
	PECVD-LF	pH 7.4	0.794	
	PECVD-LF	pH 8	1.585	
	PECVD-LF	pH 10	1.995	
	PECVD-LF	pH 12	3.981	
	PECVD-HF	pH 7.4	0.794	
	PECVD-HF	pH 8	2.512	
	PECVD-HF	pH 10	6.310	
	PECVD-HF	pH 12	25.119	
Spin-on glass (SOG) [47]	cured at 300 °C	PBS, pH 7.4	50.000	Si(OH)_4_
	cured at 800 °C	PBS, pH 7.4	6.000	

## References

[1] Dubal D.P., Chodankar N.R., Kim D.H., Gomez-Romero P. (2018). Towards flexible solid-state supercapacitors for smart and wearable electronics. Chem. Soc. Rev..

[2] Annapureddy V., Na S.M., Hwang G.T., Kang M.G., Sriramdas R., Palneedi H., Yoon W.H., Hahn B.D., Kim J.W., Ahn C.W. (2018). Exceeding milli-watt powering magneto-mechano-electric generator for standalone-powered electronics. Energy Environ. Sci..

[3] Salauddin M., Toyabur R.M., Maharjan P., Park J.Y. (2018). High performance human-induced vibration driven hybrid energy harvester for powering portable electronics. Nano Energy.

[4] Wang S.H., Xu J., Wang W.C., Wang G.J.N., Rastak R., Molina-Lopez F., Chung J.W., Niu S.M., Feig V.R., Lopez J. (2018). Skin electronics from scalable fabrication of an intrinsically stretchable transistor array. Nature.

[5] Falco A., Rivadeneyra A., Loghin F.C., Salmeron J.F., Lugli P., Abdelhalim A. (2018). Towards low-power electronics: Self-recovering and flexible gas sensors. J. Mater. Chem. A.

[6] Li R.F., Wang L., Kong D.Y., Yin L. (2018). Recent progress on biodegradable materials and transient electronics. Bioact. Mater..

[7] Gao Y., Zhang Y., Wang X., Sim K., Liu J.S., Chen J., Feng X., Xu H.X., Yu C.J. (2017). Moisture-triggered physically transient electronics. Sci. Adv..

[8] Yoon J., Lee J., Choi B., Lee D., Kim D.H., Kim D.M., Moon D.I., Lim M., Kim S., Choi S.J. (2017). Flammable carbon nanotube transistors on a nitrocellulose paper substrate for transient electronics. Nano Res..

[9] Lee G., Kang S.K., Won S.M., Gutruf P., Jeong Y.R., Koo J., Lee S.S., Rogers J.A., Ha J.S. (2017). Fully Biodegradable Microsupercapacitor for Power Storage in Transient Electronics. Adv. Energy Mater..

[10] Kang S.-K., Hwang S.-W., Cheng H., Yu S., Kim B.H., Kim J.-H., Huang Y., Rogers J.A. (2014). Dissolution Behaviors and Applications of Silicon Oxides and Nitrides in Transient Electronics. Adv. Funct. Mater..

[11] Khanra S., Cipriano T., Lam T., White T.A., Fileti E.E., Alves W.A., Guha S. (2015). Self-Assembled Peptide-Polyfluorene Nanocomposites for Biodegradable Organic Electronics. Adv. Mater. Interfaces.

[12] Feig V.R., Tran H., Bao Z.N. (2018). Biodegradable Polymeric Materials in Degradable Electronic Devices. ACS Cent. Sci..

[13] Kang S.K., Koo J., Lee Y.K., Rogers J.A. (2018). Advanced Materials and Devices for Bioresorbable Electronics. Acc. Chem. Res..

[14] Hwang S.W., Tao H., Kim D.H., Cheng H.Y., Song J.K., Rill E., Brenckle M.A., Panilaitis B., Won S.M., Kim Y.S. (2012). A Physically Transient Form of Silicon Electronics. Science.

[15] Kang S.K., Murphy R.K.J., Hwang S.W., Lee S.M., Harburg D.V., Krueger N.A., Shin J.H., Gamble P., Cheng H.Y., Yu S. (2016). Bioresorbable silicon electronic sensors for the brain. Nature.

[16] Irimia-Vladu M., Glowacki E.D., Voss G., Bauer S., Sariciftci N.S. (2012). Green and biodegradable electronics. Mater. Today.

[17] Park C.W., Kang S.K., Hernandez H.L., Kaitz J.A., Wie D.S., Shin J., Lee O.P., Sottos N.R., Moore J.S., Rogers J.A. (2015). Thermally Triggered Degradation of Transient Electronic Devices. Adv. Mater..

[18] Hernandez H.L., Kang S.K., Lee O.P., Hwang S.W., Kaitz J.A., Inci B., Park C.W., Chung S.J., Sottos N.R., Moore J.S. (2014). Triggered Transience of Metastable Poly(phthalaldehyde) for Transient Electronics. Adv. Mater..

[19] Kim D.H., Lu N.S., Ma R., Kim Y.S., Kim R.H., Wang S.D., Wu J., Won S.M., Tao H., Islam A. (2011). Epidermal Electronics. Science.

[20] Hwang S.W., Kim D.H., Tao H., Kim T.I., Kim S., Yu K.J., Panilaitis B., Jeong J.W., Song J.K., Omenetto F.G. (2013). Materials and Fabrication Processes for Transient and Bioresorbable High-Performance Electronics. Adv. Funct. Mater..

[21] Chang J.K., Chang H.P., Guo Q., Koo J., Wu C.I., Rogers J.A. (2018). Biodegradable Electronic Systems in 3D, Heterogeneously Integrated Formats. Adv. Mater..

[22] Hwang S.-W., Park G., Edwards C., Corbin E.A., Kang S.-K., Cheng H., Song J.-K., Kim J.-H., Yu S., Ng J. (2014). Dissolution Chemistry and Biocompatibility of Single-Crystalline Silicon Nanomembranes and Associated Materials for Transient Electronics. ACS Nano.

[23] Hwang S.W., Park G., Cheng H., Song J.K., Kang S.K., Yin L., Kim J.H., Omenetto F.G., Huang Y.G., Lee K.M. (2014). 25th Anniversary Article: Materials for High-Performance Biodegradable Semiconductor Devices. Adv. Mater..

[24] Yin L., Farimani A.B., Min K., Vishal N., Lam J., Lee Y.K., Aluru N.R., Rogers J.A. (2015). Mechanisms for hydrolysis of silicon nanomembranes as used in bioresorbable electronics. Adv. Mater..

[25] Dagdeviren C., Hwang S.W., Su Y.W., Kim S., Cheng H.Y., Gur O., Haney R., Omenetto F.G., Huang Y.G., Rogers J.A. (2013). Transient, Biocompatible Electronics and Energy Harvesters Based on ZnO. Small.

[26] Kang S.K., Park G., Kim K., Hwang S.W., Cheng H.Y., Shin J.H., Chung S.J., Kim M., Yin L., Lee J.C. (2015). Dissolution Chemistry and Biocompatibility of Silicon- and Germanium-Based Semiconductors for Transient Electronics. ACS Appl. Mater. Interfaces.

[27] Jin S.H., Kang S.K., Cho I.T., Han S.Y., Chung H.U., Lee D.J., Shin J., Baek G.W., Kim T.I., Lee J.H. (2015). Water-Soluble Thin Film Transistors and Circuits Based on Amorphous Indium-Gallium-Zinc Oxide. ACS Appl. Mater. Interfaces.

[28] Huang X., Liu Y.H., Hwang S.W., Kang S.K., Patnaik D., Cortes J.F., Rogers J.A. (2014). Biodegradable Materials for Multilayer Transient Printed Circuit Boards. Adv. Mater..

[29] Yin L., Cheng H., Mao S., Haasch R., Liu Y., Xie X., Hwang S.-W., Jain H., Kang S.-K., Su Y. (2014). Dissolvable Metals for Transient Electronics. Adv. Funct. Mater..

[30] Yu K.J., Kuzum D., Hwang S.W., Kim B.H., Juul H., Kim N.H., Won S.M., Chiang K., Trumpis M., Richardson A.G. (2016). Bioresorbable silicon electronics for transient spatiotemporal mapping of electrical activity from the cerebral cortex. Nat. Mater..

[31] Institute of Medicine (US) Panel on Micronutrients (2001). Dietary Reference Intakes for Vitamin A, Vitamin K, Arsenic, Boron, Chromium, Copper, Iodine, Iron, Manganese, Molybdenum, Nickel, Silicon, Vanadium, and Zinc.

[32] Ysart G., Miller P., Crews H., Robb P., Baxter M., De L’Argy C., Lofthouse S., Sargent C., Harrison N. (1999). Dietary exposure estimates of 30 elements from the UK Total Diet Study. Food Addit. Contam..

[33] Millour S., Noel L., Chekri R., Vastel C., Kadar A., Sirot V., Leblanc J.C., Guerin T. (2012). Strontium, silver, tin, iron, tellurium, gallium, germanium, barium and vanadium levels in foodstuffs from the Second French Total Diet Study. J. Food Compos. Anal..

[34] Tao S.H., Bolger P.M. (1997). Hazard assessment of germanium supplements. Regul. Toxicol. Pharm..

[35] James B., Zhang W.L., Sun P., Wu M.Y., Li H.H., Khaliq M.A., Jayasuriya P., James S., Wang G. (2017). Tungsten (W) bioavailability in paddy rice soils and its accumulation in rice (*Oryza sativa*). Int. J. Environ. Health Res..

[36] Institute of Medicine (US) Standing Committee on the Scientific Evaluation of Dietary Reference Intakes (1997). Dietary Reference Intakes for Calcium, Phosphorus, Magnesium, Vitamin D, and Fluoride.

[37] Rogers J.A., Lagally M.G., Nuzzo R.G. (2011). Synthesis, assembly and applications of semiconductor nanomembranes. Nature.

[38] Hofmann S., Ducati C., Neill R.J., Piscanec S., Ferrari A.C., Geng J., Dunin-Borkowski R.E., Robertson J. (2003). Gold catalyzed growth of silicon nanowires by plasma enhanced chemical vapor deposition. J. Appl. Phys..

[39] Guo X.L., Tabata H., Kawai T. (2001). Pulsed laser reactive deposition of p-type ZnO film enhanced by an electron cyclotron resonance source. J. Cryst. Growth.

[40] Schropp R.E.I., Feenstra K.E., Molenbroek E.C., Meiling H., Rath J.K. (1997). Device-quality polycrystalline and amorphous silicon films by hot-wire chemical vapour deposition. Philos. Mag. B.

[41] Lee W.J., Park W.T., Park S., Sung S., Noh Y.Y., Yoon M.H. (2015). Large-Scale Precise Printing of Ultrathin Sol-Gel Oxide Dielectrics for Directly Patterned Solution-Processed Metal Oxide Transistor Arrays. Adv. Mater..

[42] Kim D.H., Ghaffari R., Lu N.S., Rogers J.A. (2012). Flexible and Stretchable Electronics for Biointegrated Devices. Annu. Rev. Biomed. Eng..

[43] Ahn J.H., Kim H.S., Lee K.J., Jeon S., Kang S.J., Sun Y.G., Nuzzo R.G., Rogers J.A. (2006). Heterogeneous three-dimensional electronics by use of printed semiconductor nanomaterials. Science.

[44] Lee Y.K., Yu K.J., Song E.M., Farimani A.B., Vitale F., Xie Z.Q., Yoon Y., Kim Y., Richardson A., Luan H.W. (2017). Dissolution of Monocrystalline Silicon Nanomembranes and Their Use as Encapsulation Layers and Electrical Interfaces in Water-Soluble Electronics. ACS Nano.

[45] Chen X., Park Y.J., Kang M., Kang S.K., Koo J., Shinde S.M., Shin J., Jeon S., Park G., Yan Y. (2018). CVD-grown monolayer MoS2 in bioabsorbable electronics and biosensors. Nat. Commun..

[46] Mahajan B.K., Yu X.W., Shou W., Pan H., Huang X. (2017). Mechanically Milled Irregular Zinc Nanoparticles for Printable Bioresorbable Electronics. Small.

[47] Kang S.-K., Hwang S.-W., Yu S., Seo J.-H., Corbin E.A., Shin J., Wie D.S., Bashir R., Ma Z., Rogers J.A. (2015). Biodegradable Thin Metal Foils and Spin-On Glass Materials for Transient Electronics. Adv. Funct. Mater..

[48] Kirkland N.T., Birbilis N., Staiger M.P. (2012). Assessing the corrosion of biodegradable magnesium implants: A critical review of current methodologies and their limitations. Acta Biomater..

[49] Bowen P.K., Drelich J., Goldman J. (2013). Zinc Exhibits Ideal Physiological Corrosion Behavior for Bioabsorbable Stents. Adv. Mater..

[50] Badawy W.A., Al-Kharafi F.M. (1998). Corrosion and passivation behaviors of molybdenum in aqueous solutions of different pH. Electrochim. Acta.

[51] Patrick E., Orazem M.E., Sanchez J.C., Nishida T. (2011). Corrosion of tungsten microelectrodes used in neural recording applications. J. Neurosci. Meth..

[52] Hemstreet J.M. (1982). Dielectric constant of cotton. J. Electrost..

[53] Jayamani E., Hamdan S., Rahman M.R., Bin Bakri M.K. (2014). Comparative Study of Dielectric Properties of Hybrid Natural Fiber Composites. Procedia Eng..

[54] Irimia-Vladu M., Troshin P.A., Reisinger M., Shmygleva L., Kanbur Y., Schwabegger G., Bodea M., Schwodiauer R., Mumyatov A., Fergus J.W. (2010). Biocompatible and Biodegradable Materials for Organic Field-Effect Transistors. Adv. Funct. Mater..

[55] Singh T.B., Sariciftci N.S., Grote J.G. (2010). Bio-Organic Optoelectronic Devices Using DNA. Org. Electron..

[56] Yumusak C., Singh T.B., Sariciftci N.S., Grote J.G. (2009). Bioorganic field effect transistors based on crosslinked deoxyribonucleic acid (DNA) gate dielectric. Appl. Phys. Lett..

[57] Singh B., Sariciftci N.S., Grote J.G., Hopkins F.K. (2006). Bioorganic-semiconductor-field-effect-transistor based on deoxyribonucleic acid gate dielectric. J. Appl. Phys..

[58] Wang L., Yoshida J., Ogata N., Sasaki S., Kajiyama T. (2001). Self-Assembled Supramolecular Films Derived from Marine Deoxyribonucleic Acid (DNA)−Cationic Surfactant Complexes: Large-Scale Preparation and Optical and Thermal Properties. Chem. Mater..

[59] Worfolk B.J., Andrews S.C., Park S., Reinspach J., Liu N., Toney M.F., Mannsfeld S.C., Bao Z.N. (2015). Ultrahigh electrical conductivity in solutionsheared polymeric transparent films. Proc. Natl. Acad. Sci. USA.

[60] Qiao Y.L., Guo Y.L., Yu C.M., Zhang F.J., Xu W., Liu Y.Q., Zhu D.B. (2012). Diketopyrrolopyrrole-Containing Quinoidal Small Molecules for High-Performance, Air-Stable, and Solution-Processable n-Channel Organic Field-Effect Transistors. J. Am. Chem. Soc..

[61] Chen H.J., Guo Y.L., Yu G., Zhao Y., Zhang J., Gao D., Liu H.T., Liu Y.Q. (2012). Highly p-Extended Copolymers with Diketopyrrolopyrrole Moieties for High-Performance Field-Effect Transistors. Adv. Mater..

[62] Madrigal M.M.P., Giannotti M.I., Oncins G., Franco L., Armelin E., Puiggali J., Sanz F., del Valle L.J., Aleman C. (2013). Bioactive nanomembranes of semiconductor polythiophene and thermoplastic polyurethane: Thermal, nanostructural and nanomechanical properties. Polym. Chem..

[63] Lei T., Guan M., Liu J., Lin H.C., Pfattner R., Shaw L., McGuire A.F., Huang T.C., Shao L.L., Cheng K.T. (2017). Biocompatible and totally disintegrable semiconducting polymer for ultrathin and ultralightweight transient electronics. Proc. Natl. Acad. Sci. USA.

[64] Irimia-Vladu M., Glowacki E.D., Troshin P.A., Schwabegger G., Leonat L., Susarova D.K., Krystal O., Ullah M., Kanbur Y., Bodea M.A. (2012). Indigo—A Natural Pigment for High Performance Ambipolar Organic Field Effect Transistors and Circuits. Adv. Mater..

[65] Mostert A.B., Powell B.J., Pratt F.L., Hanson G.R., Sarna T., Gentle I.R., Meredith P. (2012). Role of semiconductivity and ion transport in the electrical conduction of melanin. Proc. Natl. Acad. Sci. USA.

[66] Bettinger C.J., Bruggeman P.P., Misra A., Borenstein J.T., Langer R. (2009). Biocompatibility of biodegradable semiconducting melanin films for nerve tissue engineering. Biomaterials.

[67] Ramachandran G.K., Tomfohr J.K., Li J., Sankey O.F., Zarate X., Primak A., Terazono Y., Moore T.A., Moore A.L., Gust D. (2003). Electron transport properties of a carotene molecule in a metal-(single molecule)-metal junction. J. Phys. Chem. B.

[68] Koh L.D., Yeo J., Lee Y.Y., Ong Q., Han M.Y., Tee B.C.K. (2018). Advancing the frontiers of silk fibroin protein-based materials for futuristic electronics and clinical wound-healing. Mater. Sci. Eng. C Mater..

[69] Li G., Li Y., Chen G.Q., He J.H., Han Y.F., Wang X.Q., Kaplan D.L. (2015). Silk-Based Biomaterials in Biomedical Textiles and Fiber-Based Implants. Adv. Healthc. Mater..

[70] Xie M.B., Li Y., Li J.S., Chen A.Z., Zhao Z., Li G. (2014). Biomedical Applications of Silk Fibroin. Textile Bioengineering and Informatics Symposium Proceedings.

[71] Taddei P., Chiono V., Anghileri A., Vozzi G., Freddi G., Ciardelli G. (2013). Silk Fibroin/Gelatin Blend Films Crosslinked with Enzymes for Biomedical Applications. Macromol. Biosci..

[72] Pal R.K., Farghaly A.A., Wang C.Z., Collinson M.M., Kundu S.C., Yadavalli V.K. (2016). Conducting polymer-silk biocomposites for flexible and biodegradable electrochemical sensors. Biosens. Bioelectron..

[73] Edupuganti V., Solanki R. (2016). Fabrication, characterization, and modeling of a biodegradable battery for transient electronics. J. Power Sources.

[74] Jiang L., Zhang J. (2017). Biodegradable and biobased polymers. Applied Plastics Engineering Handbook.

[75] Zhu H.L., Fang Z.Q., Preston C., Li Y.Y., Hu L.B. (2014). Transparent paper: Fabrications, properties, and device applications. Energy Environ. Sci..

[76] Zhu H.L., Xiao Z.G., Liu D.T., Li Y.Y., Weadock N.J., Fang Z.Q., Huang J.S., Hu L.B. (2013). Biodegradable transparent substrates for flexible organic-light-emitting diodes. Energy Environ. Sci..

[77] Jung Y.H., Chang T.H., Zhang H.L., Yao C.H., Zheng Q.F., Yang V.W., Mi H.Y., Kim M., Cho S.J., Park D.W. (2015). High-performance green flexible electronics based on biodegradable cellulose nanofibril paper. Nat. Commun..

[78] Li Z.Q., Wang H.C., Lv S.Z., Liu L., Guo W.Y., Yuan M., Yan H.B., Zhao H.J., Lang S.P. (2011). Clinical Comparative Study on Efficacy and Safety for Treatment of Coronary Heart Disease with Cobalt-Base Alloy Bio Absorbable Polymer Sirolimus-Eluting Stent and Partner Stent. Heart.

[79] Wu Y.T., Gao Y.C. (2013). Five Years Follow Up Result after Application of Biodegradable Polymer Sirolimus-Eluting Stent in Patients with Coronary Heart Disease and Diabetes Mellitus. Heart.

[80] Inigo-Garcia L.A., Martinez-Garcia F.J., Milan-Pinilla A., Valle-Alberca A., Fernandez-Lopez L., Traverso-Castilla V.V., Delgado-Aguilar A., Bravo-Marques R., Ramirez-Moreno A., Siles-Rubio J.R. (2016). Biodegradable Polymer Drug Eluting Stent: Efficacy and Safety with Short Regimen of Antiplatelet Therapy. Cardiology.

[81] Pilgrim T., Heg D., Roffi M., Tuller D., Muller O., Vuilliomenet A., Cook S., Weilenmann D., Kaiser C., Jamshidi P. (2014). Ultrathin strut biodegradable polymer sirolimus-eluting stent versus durable polymer everolimus-eluting stent for percutaneous coronary revascularisation (BIOSCIENCE): A randomised, single-blind, non-inferiority trial. Lancet.

[82] Waksman R., Pakala R., Baffour R., Seabron R., Hellinga D., Chan R., Su S.H., Kolodgie F., Virmani R. (2012). In vivo comparison of a polymer-free Biolimus A9-eluting stent with a biodegradable polymer-based Biolimus A9 eluting stent and a bare metal stent in balloon denuded and radiated hypercholesterolemic rabbit iliac arteries. Catheter. Cardiovasc. Interv..

[83] Balch O.K., Collier M.A., DeBault L.E., Johnson L.L. (1999). Bioabsorbable suture anchor (co-polymer 85/15 D,L lactide/glycolide) implanted in bone: Correlation of physical/mechanical properties, magnetic resonance imaging, and histological response. Arthroscopy.

[84] Im S.H., Jung Y., Kim S.H. (2017). Current status and future direction of biodegradable metallic and polymeric vascular scaffolds for next-generation stents. Acta Biomater..

[85] Hwang S.W., Song J.K., Huang X., Cheng H.Y., Kang S.K., Kim B.H., Kim J.H., Yu S., Huang Y.G., Rogers J.A. (2014). High-Performance Biodegradable/Transient Electronics on Biodegradable Polymers. Adv. Mater..

[86] Shou W., Mahajan B.K., Ludwig B., Yu X.W., Staggs J., Huang X., Pan H. (2017). Low-Cost Manufacturing of Bioresorbable Conductors by Evaporation-Condensation-Mediated Laser Printing and Sintering of Zn Nanoparticles. Adv. Mater..

[87] Najafabadi A.H., Tamayol A., Annabi N., Ochoa M., Mostafalu P., Akbari M., Nikkhah M., Rahimi R., Dokmeci M.R., Sonkusale S. (2014). Biodegradable Nanofibrous Polymeric Substrates for Generating Elastic and Flexible Electronics. Adv. Mater..

[88] Boutry C.M., Kaizawa Y., Schroeder B.C., Chortos A., Legrand A., Wang Z., Chang J., Fox P., Bao Z. (2018). A stretchable and biodegradable strain and pressure sensor for orthopaedic application. Nat. Electron..

[89] Lee Y.K., Kim J., Kim Y., Kwak J.W., Yoon Y., Rogers J.A. (2017). Room Temperature Electrochemical Sintering of Zn Microparticles and Its Use in Printable Conducting Inks for Bioresorbable Electronics. Adv. Mater..

[90] Brenckle M.A., Cheng H.Y., Hwang S., Tao H., Paquette M., Kaplan D.L., Rogers J.A., Huang Y.G., Omenetto F.G. (2015). Modulated Degradation of Transient Electronic Devices through Multilayer Silk Fibroin Pockets. ACS Appl. Mater. Interfaces.

[91] Pan R.Z., Xuan W.P., Chen J.K., Dong S.R., Jin H., Wang X.Z., Li H.L., Luo J.K. (2018). Fully biodegradable triboelectric nanogenerators based on electrospun polylactic acid and nanostructured gelatin films. Nano Energy.

[92] Lee S., Koo J., Kang S.K., Park G., Lee Y.J., Chen Y.Y., Lim S.A., Lee K.M., Rogers J.A. (2018). Metal microparticle—Polymer composites as printable, bio/ecoresorbable conductive inks. Mater. Today.

[93] Lee C.H., Kim H., Harburg D.V., Park G., Ma Y.J., Pan T.S., Kim J.S., Lee N.Y., Kim B.H., Jang K.I. (2015). Biological lipid membranes for on-demand, wireless drug delivery from thin, bioresorbable electronic implants. Npg Asia Mater..

[94] White M.D., Bosio C.M., Duplantis B.N., Nano F.E. (2011). Human body temperature and new approaches to constructing temperature-sensitive bacterial vaccines. Cell. Mol. Life Sci..

[95] Duplantis B.N., Bosio C.M., Nano F.E. (2011). Temperature-sensitive bacterial pathogens generated by the substitution of essential genes from cold-loving bacteria: Potential use as live vaccines. J. Mol. Med..

[96] Hooke A.M. (1994). Temperature-Sensitive Mutants of Bacterial Pathogens—Isolation and Use to Determine Host Clearance and In-Vivo Replication Rates. Method Enzymol..

[97] Tao H., Hwang S.W., Marelli B., An B., Moreau J.E., Yang M.M., Brenckle M.A., Kim S., Kaplan D.L., Rogers J.A. (2014). Silk-based resorbable electronic devices for remotely controlled therapy and in vivo infection abatement. Proc. Natl. Acad. Sci. USA.

[98] Yin L., Huang X., Xu H.X., Zhang Y.F., Lam J., Cheng J.J., Rogers J.A. (2014). Materials, Designs, and Operational Characteristics for Fully Biodegradable Primary Batteries. Adv. Mater..

[99] Bouhlala M.A., Kameche M., Tadji A., Benouar A. (2018). Chitosan hydrogel-based electrolyte for clean and biodegradable batteries: Energetic and conductometric studies. Phys. Chem. Liq..

[100] Jia X.T., Wang C.Y., Ranganathan V., Napier B., Yu C.C., Chao Y.F., Forsyth M., Omenetto F.G., MacFarlane D.R., Wallace G.G. (2017). A Biodegradable Thin-Film Magnesium Primary Battery Using Silk Fibroin-Ionic Liquid Polymer Electrolyte. ACS Energy Lett..

[101] Jia X.T., Wang C.Y., Zhao C., Ge Y., Wallace G.G. (2016). Toward Biodegradable Mg-Air Bioelectric Batteries Composed of Silk Fibroin-Polypyrrole Film. Adv. Funct. Mater..

[102] Huang X.Y., Wang D., Yuan Z.Y., Xie W.S., Wu Y.X., Li R.F., Zhao Y., Luo D., Cen L., Chen B.B. (2018). A Fully Biodegradable Battery for Self-Powered Transient Implants. Small.

[103] Lu L.Y., Yang Z.J., Meacham K., Cvetkovic C., Corbin E.A., Vazquez-Guardado A., Xue M.T., Yin L., Boroumand J., Pakeltis G. (2018). Biodegradable Monocrystalline Silicon Photovoltaic Microcells as Power Supplies for Transient Biomedical Implants. Adv. Energy Mater..

[104] Hwang S.W., Huang X., Seo J.H., Song J.K., Kim S., Hage-Ali S., Chung H.J., Tao H., Omenetto F.G., Ma Z.Q. (2013). Materials for Bioresorbable Radio Frequency Electronics. Adv. Mater..

